# Healthy Lifestyle, multimorbidity network, and all-cause mortality among older Chinese: a longitudinal analysis in Chinese longitudinal healthy longevity survey

**DOI:** 10.1186/s12889-026-26294-8

**Published:** 2026-01-16

**Authors:** Yilin Chen, Huan Zhou, Siqing Wang, Lingqiu Dong, Yi Tang, Jiaxing Tan, Wei Qin

**Affiliations:** Division of Nephrology, Department of Medicine, West China Hospital, Sichuan University, Chengdu, Sichuan China, People’s Republic

**Keywords:** Healthy lifestyle, Older people, Multimorbidity, Comorbidity network, Longitudinal study

## Abstract

**Background:**

With population ageing, multimorbidity has become a major public health concern. Although healthy lifestyles are associated with reduced risks of single chronic diseases and mortality, their relationship with multimorbidity patterns among older Chinese remains insufficiently explored.

**Methods:**

Data from 16,820 participants aged 60 and older, from the 2008–2018 waves of the China Longitudinal Healthy Longevity Survey (CLHLS), were analyzed. Participants were categorized into three lifestyle groups (favorable, average, and unfavorable) based on five modifiable lifestyle factors: social engagement, physical activity, smoking, drinking, and diet. Disease progression was assessed using multimorbidity networks, and all-cause mortality was analyzed with Cox proportional hazards models.

**Results:**

Compared to an unfavorable lifestyle, a favorable lifestyle was associated with a lower risk of all-cause mortality (HR = 1.63, 95% CI: 1.50–1.77). Among the five lifestyle factors, social engagement showed the strongest association with mortality (HR for inactive vs. active engagement = 1.36, 95% CI: 1.29–1.43). Multimorbidity networks revealed that individuals with favorable lifestyles exhibited denser, more interconnected disease networks, while those with unfavorable lifestyles showed more streamlined, linear disease progressions, primarily driven by hypertension, cardiovascular disease, and dementia.

**Conclusions:**

A favorable lifestyle was linked to lower mortality and more complex multimorbidity networks, likely due to longer survival and the accumulation of chronic conditions. These findings underscore the need for interventions to reduce premature mortality and manage multimorbidity in aging populations.

**Supplementary Information:**

The online version contains supplementary material available at 10.1186/s12889-026-26294-8.

## Introduction

The rising prevalence of multimorbidity—the co-occurrence of two or more chronic conditions—poses a major challenge to healthcare systems amidst global population ageing [1–4]. Understanding the complex interrelationships among these conditions, rather than viewing them in isolation, is crucial. Network analysis has emerged as a powerful tool to elucidate such comorbidity patterns and disease progression pathways in older populations.For example, network approaches have been applied to map frailty components, identify central diseases in multimorbidity clusters, and reveal temporal sequences in chronic disease development [5–7]. However, how modifiable lifestyle factors collectively influence the structure and evolution of these multimorbidity networks remains largely unexplored.

The relationship between lifestyle factors and health outcomes in older adults has garnered increasing attention. Research has shown that lifestyle choices—such as physical activity, diet, smoking, and social engagement—can significantly influence mortality and chronic disease risk [8–12]. However, most existing studies have focused on individual lifestyle factors and their effects on disease outcomes, often overlooking the complex interplay of multiple lifestyle factors and the dynamic nature of disease progression over time. Moreover, previous research has been limited by cross-sectional designs and by focusing on a narrow set of diseases, often neglecting the complexity of multimorbidity [13, 14].

This study aims to fill these gaps by utilizing longitudinal data from the Chinese Longitudinal Healthy Longevity Survey (CLHLS) to examine the combined impact of multiple modifiable lifestyle factors on disease progression and all-cause mortality in older Chinese adults. With China’s aging population and the growing burden of chronic diseases, our research is timely and essential for informing public health policies that promote healthy aging.

## Methods

### Study design and population

The CLHLS is a large, prospective cohort study conducted in 22 provinces investigating influential factors pertaining to the health of the older people in China.The CLHLS has been previously discussed in detail [15–17].Briefly, CLHLS was initiated in 1998, with subsequent follow-up assessments every 2 to 3 years. To fill significant reductions considering loss to follow-up and death, new participants with similar characteristics were recruited at each follow-up assessment. The survey collected information on sociodemographic characteristics, family structure, self-reported health status, quality of life, cognitive function, psychological status, activities of daily living(ADL), and lifestyle factors.In addition, dates and causes of death were obtained from family members of deceased participants.

For the present analysis, we used data from the 2008–2018 waves of the CLHLS. Participants younger than 60 years were excluded. Patterns of missing data are shown in Supplementary Fig. 1. Missing values were imputed using multiple imputation by chained equations (MICE) with the mice package in R. After exclusions and imputations, a total of 16,820 participants were included (Supplementary Fig. 2). Participants were followed from baseline until death, loss to follow-up, or the end of the study on July 31, 2019, whichever came first.

### Assessment of lifestyle factors

Five modifiable lifestyle factors—social engagement, physical activity, smoking, drinking, and diet—were assessed using self-reported information collected through structured interviews, following definitions adopted in previous studies [18].Participants who reported exercising regularly, defined as at least twice per week, were categorized as having a healthy level of physical activity. Dietary habits were evaluated based on the frequency of consumption of twelve food groups (vegetables, fruits, meat, fish, eggs, beans, tea, garlic, milk, nuts, mushrooms, and algae), with a healthy diet defined as the regular intake of at least seven of these groups. Smoking and drinking status were each classified into three categories: never, former, and current. For both behaviors, only never users were considered to have a healthy status; former users were classified as unhealthy based on evidence that cessation in older adults often reflects health deterioration rather than voluntary health promotion [19]. Social engagement was assessed through participation in six types of activities, including gardening, reading, raising domestic animals or pets, playing cards or mah-jongg, watching television or listening to the radio, and taking part in organized social activities; active engagement was defined as participating in at least two of these activities weekly. Each lifestyle factor was evaluated individually in relation to prognosis, and participants were further categorized into three groups according to the total number of healthy factors: favorable (4–5), average (2–3), and unfavorable (0–1).

### Assessment of chronic conditions

Information on 22 chronic conditions was collected in the CLHLS. Three conditions with a prevalence of less than 1% in 2018—epilepsy, breast disease, and uterine tumor—were excluded from the present analysis (Supplementary Fig. 3). Nineteen chronic conditions were therefore included: hypertension, diabetes, heart disease, stroke/cerebrovascular disease, respiratory diseases (chronic bronchitis, emphysema, pneumonia, or asthma), tuberculosis, cataract, glaucoma, cancer, prostate tumor, peptic ulcer (gastric or duodenal ulcer), parkinson’s disease, bedsore, arthritis, dementia, biliary tract diseases (cholecystitis or cholelithiasis), blood disease, chronic nephritis, and hepatitis.

### Covariates

A wide range of covariates were considered based on previous literature and clinical experience. Sociodemographic variables included age (60–69, 70–79, 80–89, and ≥ 90 years), sex (female/male), residence (rural/urban), marital status (married and living with spouse/separated, divorced, widowed, or unmarried), and education (≥ 1 year of schooling/illiterate). Functional status was assessed using ADL; the inability to perform any of the six ADL tasks—bathing, dressing, toileting, transferring, eating, and continence—was defined as disability.

### Statistical analysis

Baseline characteristics were summarized as means and standard deviations(SDs) for continuous variables and as counts and percentages for categorical variables. Group differences across lifestyle categories were assessed using the Kruskal–Wallis test (continuous variables) and the χ² test (categorical variables).

To evaluate the association between lifestyle and all-cause mortality, Kaplan–Meier curves with log-rank tests were used to compare survival across lifestyle groups. Multivariable Cox proportional hazards models were then fitted to estimate hazard ratios (HRs) and 95% confidence intervals (CIs), adjusting for age, sex, residence, education, marital status, annual income, and disability status at baseline.

To characterize multimorbidity patterns, we constructed disease networks from 19 chronic conditions. For each pair of conditions, we computed measures of comorbidity intensity: the relative risk (RR) of co-occurrence and the Pearson correlation. Pairs were considered for downstream analysis if at least 0.1% of participants had both conditions. Multiple testing was controlled using the Benjamini–Hochberg procedure; pairs with RR > 1, positive correlation and a false discovery rate (FDR)–adjusted P value < 0.05 were retained. We further examined population-level temporal ordering between disease pairs.Among participants who had both conditions and non-tied onset categories, we applied one-sided binomial tests to assess whether condition A preceded condition B more often than the reverse; P values were FDR-adjusted. For pairs with a predominant ordering, we evaluated consistency with covariate-adjusted logistic regression models. Directions were retained when the ordering-consistent odds ratio(OR) exceeded 1 with FDR-adjusted *P* < 0.05; otherwise, the pair was treated as undirected. A more detailed description of this method is provided in Supplemental Methods.

All analyses were performed in R version 4.3.2, and networks were visualized in Cytoscape version 3.8.2.

### Sensitivity analysis

Several sensitivity analyses were conducted to test the robustness of our findings.First, we repeated the Cox proportional hazards models after excluding participants who died within the first two years of follow-up to reduce the potential influence of reverse causation.Second, we stratified analysis by sex to examine potential heterogeneity in multimorbidity patterns.

## Results

### Baseline characteristics

Table 1 presents the baseline characteristics of the study population stratified by lifestyle groups.Compared with the unfavorable lifestyle group, participants with a favorable lifestyle were generally younger, with 20% aged 60–70 years versus 12% in the unfavorable group, while the latter had a much higher proportion aged over 90 years (40% vs. 25%, *p* < 0.001).The favorable group also had a higher proportion of urban residents, a markedly lower prevalence of illiteracy, and more were currently married and living with a spouse. In terms of socioeconomic and functional status, 85% of the favorable group reported an annual income above 5,000 yuan compared with 61% in the unfavorable group, and only 11% had disabilities.

**Table 1:** Baseline characteristics of CLHLS participants

Characteristic	Lifestyle group		
	Favourable	Average	Unfavourable
Number of participants	2,731	12,127	1,962
Age			
60–70 years old	555 (20%)	1,172 (9.7%)	237 (12%)
70–80 years old	791 (29%)	1,927 (16%)	327 (17%)
80–90 years old	713 (26%)	3,226 (27%)	610 (31%)
> 90 years old	672 (25%)	5,802 (48%)	788 (40%)
Male,%	1,204 (44%)	5,082 (42%)	865 (44%)
Urban,%	1,723 (63%)	4,382 (36%)	530 (27%)
Illiterate,%	1,052 (39%)	8,310 (69%)	1,141 (58%)
Currently married and living with spouse,%	1,273 (47%)	3,182 (26%)	689 (35%)
Annual income (yuan)			
< 3000	175 (6.4%)	1,824 (15%)	369 (19%)
3000–5000	241 (8.8%)	2,024 (17%)	393 (20%)
> 5000	2,315 (85%)	8,279 (68%)	1,200 (61%)
With disabilities,%	299 (11%)	3,111 (26%)	313 (16%)
Socially inactive,%	453 (17%)	9,414 (78%)	1,729 (88%)
Current exerciser,%	2,204 (81%)	2,291 (19%)	112 (5.7%)
Ever smoker,%	149 (5.5%)	1,353 (11%)	1,412 (72%)
Ever drinker,%	178 (6.5%)	1,316 (11%)	1,387 (71%)
Unfavourable diet,%	683 (25%)	9,693 (81%)	1,765 (94%)
Multimorbidity at baseline,%	1,448 (48%)	9,749 (80%)	1,867 (95%)

Values are presented as n (%) unless otherwise indicated. P values were calculated using the Chi-square test for categorical variables and the Kruskal-Wallis test for continuous variables

### Lifestyle factors and mortality

Kaplan–Meier survival curves (Supplementary Fig. 4) demonstrated a clear gradient in survival across the three lifestyle groups. Participants with a favorable lifestyle had the highest probability of survival, whereas those with an unfavorable lifestyle had the lowest (log-rank *p* < 0.001).In multivariable Cox proportional hazards models adjusted for age, sex, residence, education, marital status, income, and disability status, both average and unfavorable lifestyles were associated with significantly elevated risks of all-cause mortality compared with a favorable lifestyle (Table 2). At the overall population level, individuals with an average lifestyle had a 37% higher risk of mortality, while those with an unfavorable lifestyle had an 63% higher risk (HR = 1.63, 95% CI: 1.50–1.77).

**Table 2:** Associations between different lifestyles and all-cause mortality of different age groups

Cox proportional-hazards model^a^			
	Exposure	HR (95%CI)	*P* value
All-cause mortality	Favourable	1.0	
	Average	1.37(1.28–1.46)	< 0.001
	Unfavourable	1.63(1.50–1.77)	< 0.001

^a^Model was adjusted for age, sex, residence, education, marital status, income, disability at baseline

When the five lifestyle factors were examined individually (Table 3), inactive social engagement, non-current exercise, and unfavorable diet showed the strongest associations with increased all-cause mortality. At the overall level, inactive social engagement was associated with a 36% higher risk of mortality (HR = 1.36, 95% CI: 1.29–1.43), non-current exercise with a 16% higher risk (HR = 1.16, 95% CI: 1.11–1.22), and an unfavorable diet with a 20% higher risk (HR = 1.20, 95% CI: 1.14–1.26). Ever smoking (including both current and former smokers) was associated with a 15% higher mortality risk compared to never smoking (HR = 1.15, 95% CI: 1.09–1.21), while ever drinking showed a more modest association (HR = 1.07, 95% CI: 1.01–1.13). All models were adjusted for age, sex, residence, education, marital status, income, disability status, and the other four lifestyle factors.

**Table 3:** Associations between five healthy lifestyle factors and all-cause mortality

	Exposure	HR (95%CI)
All-cause mortality	Social engagement(Inactive vs. Active)	1.36 (1.29–1.43)
	Exercise(Non-Current exerciser vs. Current exerciser)	1.16 (1.11–1.22)
	Smoking(Ever smoker vs. Never smoker)	1.15 (1.09–1.21)
	Drinking(Ever drinker vs. Never drinker)	1.07 (1.01–1.13)
	Diet(Unfavourable vs. favourable)	1.20 (1.14–1.26)

Model was adjusted for age, sex, residence, education, marital status, income, state of disability and other 4 factors at baseline

### Multimorbidity networks with temporal ordering

**Fig. 1:**
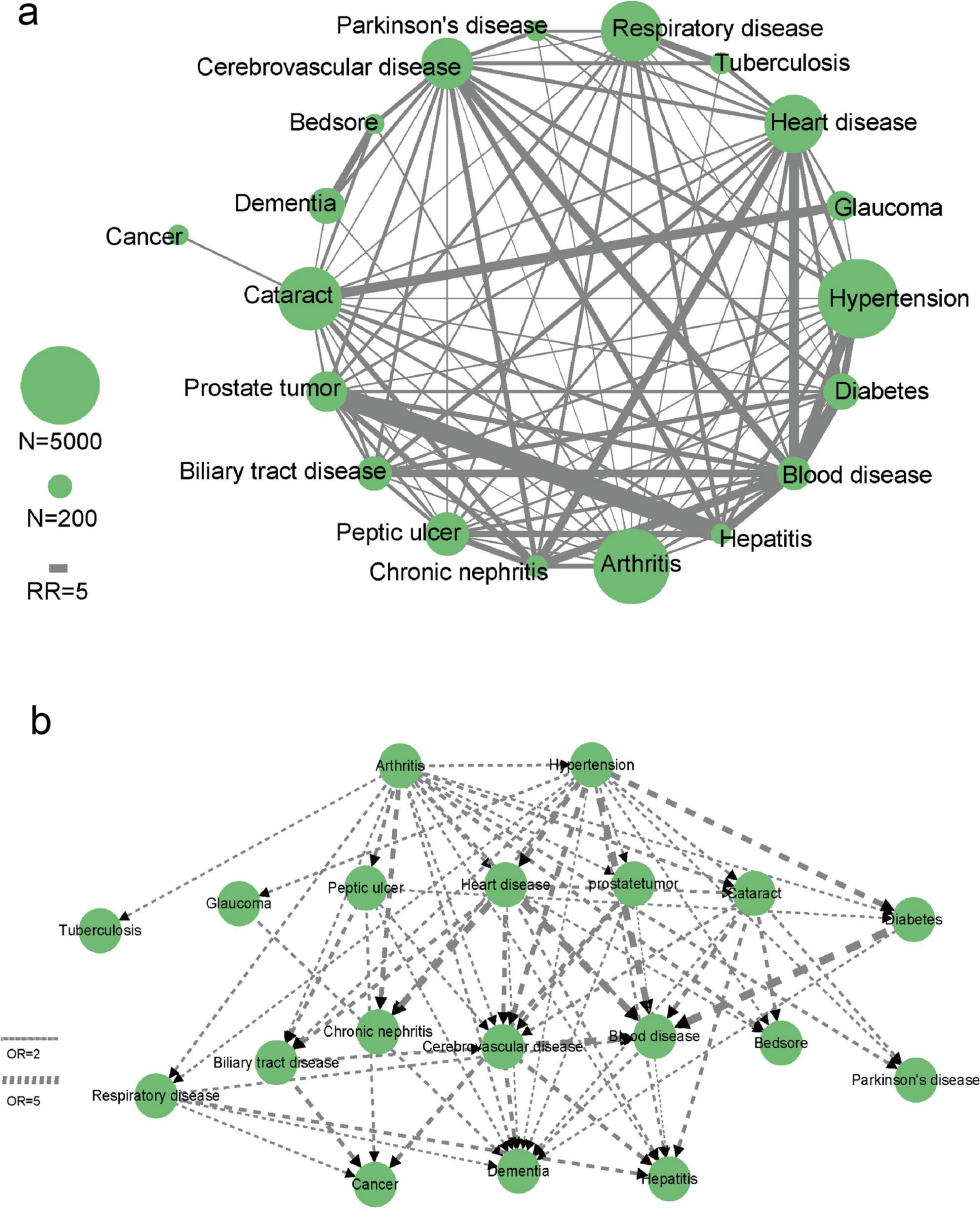
Overall multimorbidity patterns among older Chinese adults. **a** Undirected multimorbidity network of 19 chronic diseases. Nodes represent diseases, with size proportional to prevalence. Edges indicate significant comorbidity associations, with thickness proportional to relative risk. **b** Directed disease trajectory network based on temporal sequence analyses. Arrows indicate the predominant order of disease occurrence between disease pairs, and edge thickness corresponds to odds ratios from logistic regression models

Figure 1 displays the overall multimorbidity patterns among older adults. In the undirected comorbidity network (Fig. 1a), hypertension, arthritis, and cataract appeared as highly prevalent and central conditions, with strong connections to multiple other diseases. The temporally ordered network (Fig. 1b) further highlighted hypertension and cerebrovascular disease as key nodes in the population-level progression, with subsequent links to dementia and other chronic conditions.

**Fig. 2:**
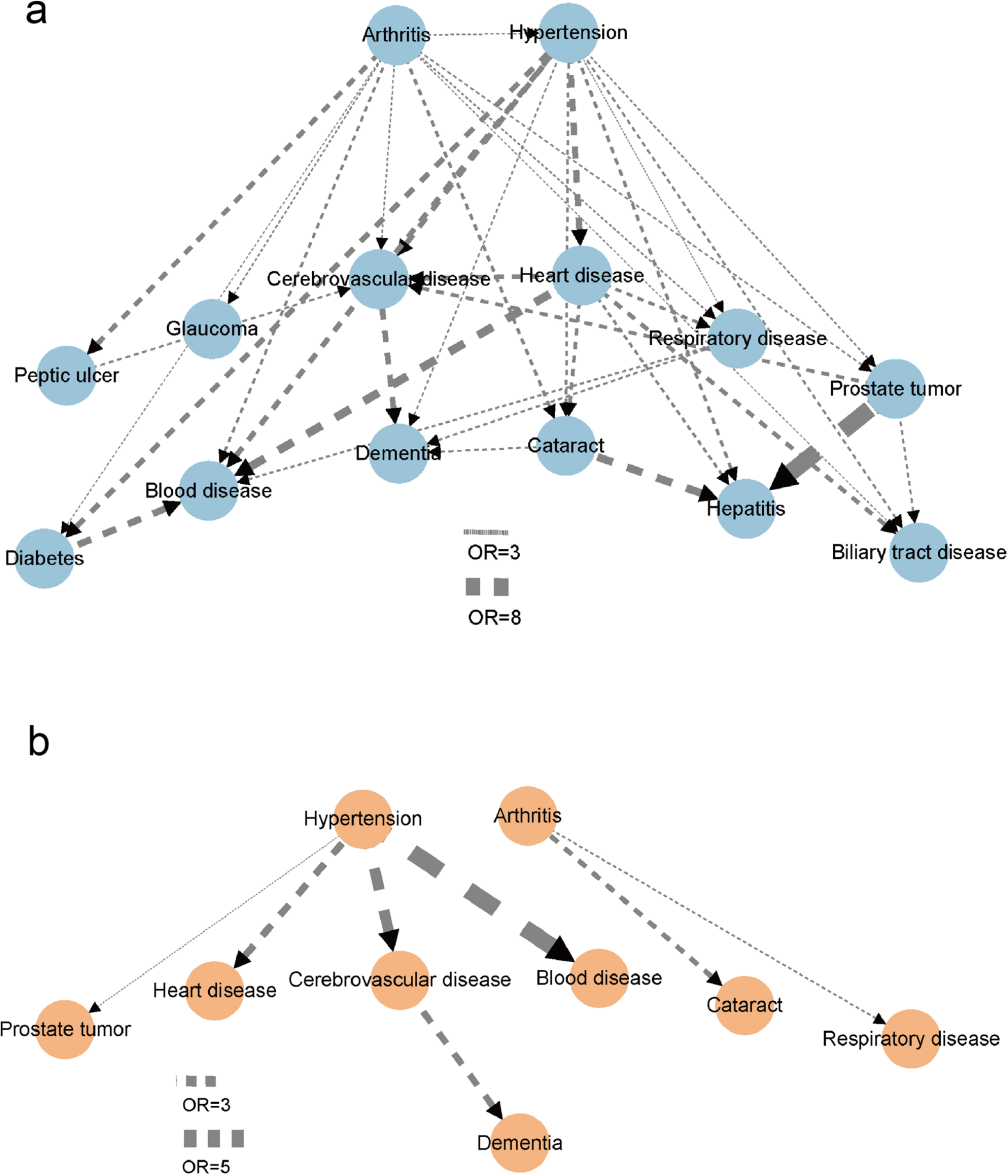
Disease trajectory networks stratified by lifestyle groups. **a** Directed network among participants with a favorable lifestyle (4–5 healthy factors). **b** Directed network among participants with an unfavorable lifestyle (0–1 healthy factors). Nodes represent diseases, with size proportional to prevalence in each group. Arrows indicate the predominant temporal order of disease occurrence, and edge thickness reflects odds ratios from logistic regression models

When stratified by lifestyle groups (Fig. 2), differences in network structures were observed. In the favourable lifestyle group (Fig. 2a), the network was denser, with a greater number of nodes and edges, and stronger interconnections among conditions such as hypertension, arthritis, and cataract (Supplementary Table 1). In contrast, the unfavourable lifestyle group (Fig. 2b) presented a simpler structure with fewer nodes and edges, in which hypertension served as the main starting point and was directly linked to heart disease, cerebrovascular disease, blood disease, and dementia. Node-level centrality measures further highlighted hypertension and arthritis as the dominant hub nodes in the favourable group, with the highest outdegree and weighted outdegree. In the unfavourable group, hypertension remained the leading hub, followed by arthritis, whereas other conditions such as cerebrovascular disease, dementia, and blood disease showed limited outdegree (Supplementary Table 2).

### Sensitivity analyses

Several sensitivity analyses were conducted to test the robustness of our findings. First, excluding participants who died within the first two years of follow-up yielded similar associations between lifestyle groups and all-cause mortality, suggesting that reverse causation was unlikely to fully explain the results(Supplementary Table 3;Supplementary Table 4). Second, when stratified by sex, multimorbidity patterns varied between males and females(Supplementary Fig. 5). In both male and female participants, the favourable lifestyle group exhibited denser networks with more interconnected diseases, reflecting a broader spectrum of multimorbidity. In contrast, the unfavourable lifestyle group showed more streamlined, linear disease progressions, with fewer interconnections, indicating a faster and simpler disease trajectory(Supplementary Fig. 6).

## Discussion

This large prospective cohort study of more than 16,000 older Chinese adults revealed two key findings. First, adherence to healthier lifestyle behaviors was consistently associated with lower all-cause mortality risk, reinforcing the critical role of modifiable lifestyle factors in promoting longevity. Second, network-based analyses of multimorbidity showed distinct structural differences by lifestyle group. Participants with an unfavorable lifestyle exhibited a more streamlined, linear disease progression dominated by hypertension, heart disease, cerebrovascular disease, and dementia, suggesting rapid and fatal pathways. By contrast, those with a favorable lifestyle displayed denser and more interconnected multimorbidity networks, which may reflect longer survival and the accumulation of multiple chronic conditions rather than accelerated fatal progression.

The relationship between lifestyle factors and health outcomes has been the subject of previous research, the majority of which concentrated on specific lifestyle factors like smoking, diet or physical exercise [20–23]. Few research have comprehensively examined the combined effects of multiple lifestyle factors on mortality risk. We found that social engagement had the strongest impact on all-cause mortality, followed by diet, physical exercise, and smoking. Social engagement helps reduce mortality by improving psychological well-being and promoting physical activity, both of which mitigate chronic disease effects. Positive social connections also buffer against stress by reducing inflammation, a key factor in age-related diseases. The impact of social engagement varies across cultures, with family ties being particularly important in Asian populations and structured community activities in Western ones. However, reverse causality must be considered, as declining health may lead to social isolation, further worsening health outcomes [24–26].The minimal impact of current drinking status on mortality risk observed in our study may be attributed to unmeasured factors, such as the quantity, type, and pattern of alcohol consumption. Previous research suggests that moderate alcohol consumption, particularly wine, may be beneficial for cardiovascular health, whereas heavy drinking increases mortality risk [27]. Moreover, the type of alcohol and drinking patterns (e.g., binge drinking) significantly influence health outcomes [28, 29]. The lack of differentiation in these aspects may have diluted the observed relationship between alcohol consumption and mortality risk in the current analysis.

Our findings extend previous studies establishing associations between a healthy lifestyle and reduced mortality in an older population, and we delineate disease networks in older adults with different lifestyles. Previous studies have primarily examined individual risk factors such as smoking, physical activity, or diet, or have focused on mortality alone. Fewer investigations have explored multimorbidity structures, particularly in the context of lifestyle heterogeneity. Recent research using UK Biobank data also suggests that lifestyle behaviors impact multimorbidity clusters [30], but it does not address the complexity of disease networks or the potential survival-related patterns in these associations.By applying network approaches, our study highlights the nuanced ways in which lifestyle shapes not only the occurrence of diseases but also their interconnections. The observation that favorable lifestyle groups showed more complex networks contrasts with the common assumption that healthier lifestyles always equate to fewer diseases, and underscores the importance of considering survival bias when interpreting multimorbidity in long-lived populations. The streamlined trajectories in the unfavorable lifestyle group suggest that high-risk behaviors accelerate progression along well-established cardiometabolic–neurological pathways, leading more quickly to mortality [31]. In contrast, participants with healthier lifestyles lived longer and therefore accumulated a wider variety of comorbid conditions, which appeared as more interconnected networks. This phenomenon reflects the concept of “survival bias” where individuals who live longer have more opportunities to develop multiple conditions [32]. From a public health perspective, these findings highlight two complementary priorities: Reducing premature mortality by targeting modifiable lifestyle risks and managing the multimorbidity burden among longer-lived individuals through integrated care models that address the complexity of multiple coexisting diseases.

Key strengths of our study include the use of a large, nationally representative cohort, long-term follow-up, and the integration of lifestyle, mortality, and multimorbidity network analyses. Nevertheless, our investigation has several limitations. First, the evaluation of lifestyle factors and disease diagnoses was based on self-reported data, which may introduce measurement errors and misclassification. Our assessment of physical activity was based on self-reported current exercise status, which categorized participants as current exercisers or non-current exercisers. This binary classification may not capture important nuances of physical activity behavior in older adults, such as exercise frequency, intensity, duration, or type. The modest association we observed (HR = 1.16 for non-current vs. current exercisers) may partly reflect this measurement limitation. Future studies with more detailed physical activity assessments are warranted to better understand its role in multimorbidity networks among older populations.Second, the limited categorization of diseases in the database, with fewer conditions included, hindered our ability to assess conditions with lower prevalence. This may have led to an underestimation of the prevalence of chronic diseases, especially considering that many older people may not seek medical attention regularly. Third, the uneven distribution of participants in the lifestyle groups could potentially affect the generalizability of our findings. However, the sample size remains adequate to ensure statistical stability. Additionally, while the multimorbidity networks were constructed at the population level and capture comorbidity patterns and potential temporal sequences, they do not represent individual-level disease trajectories. Survival bias may also partly explain the more complex networks observed in the favorable lifestyle group. Finally, despite adjusting for a wide range of covariates, residual confounding cannot be entirely excluded.

## Conclusion

In conclusion, unfavorable lifestyles were associated with higher mortality and more linear disease pathways, whereas favorable lifestyles were linked to greater longevity but more complex multimorbidity networks. These results highlight the dual importance of promoting healthy lifestyles to prevent premature death and preparing healthcare systems to address the multimorbidity burden in aging populations.

## Supplementary Information

Supplementary Material 1. Supplementary Table 1.docx Global network metrics stratified by lifestyle group. Supplementary Table 2.docx Node-level centrality measures for key diseases in favourable and unfavourable lifestyle groups. Supplementary Table 3.docx Associations between different lifestyles and all-cause mortality of different age groups after excluding participants who died within the first two years of follow-up. Supplementary Table 4.docx Associations between five healthy lifestyle factors and all-cause mortality of different age groups after excluding participants who died within the first two years of follow-up. Supplementary Figure 1.png The proportion of missing data in the variable. Supplementary Figure 2.png Flowchart for disease trajectory network analyses. Supplementary Figure 3.png Prevalence of 22 chronic medical conditions in 2018. Supplementary Figure 4.png Kaplan–Meier survival curves stratified by lifestyle. Supplementary Figure 5.png Sensitivity analysis of multimorbidity patterns stratified by sex. Supplementary Figure 6.png Sensitivity analysis of multimorbidity networks stratified by sex and lifestyle groups

## Data Availability

All related data are accessible to the public and can be obtained at https://opendata.pku.edu.cn/dataverse/CHADS.
