# Supplementary material for: Healthy Lifestyle, multimorbidity network, and all-cause mortality among older Chinese: a longitudinal analysis in Chinese longitudinal healthy longevity survey
Source: BMC Public Health. 2026 Jan 16;26:556. doi: 10.1186/s12889-026-26294-8 (PMC12892668; doi:10.1186/s12889-026-26294-8)
Supplement: Supplementary file 1 — Supplementary Material 1. Supplementary Table 1.docx Global network metrics stratified by lifestyle group. Supplementary Table 2.docx Node-level centrality measures for key diseases in favourable and unfavourable lifestyle groups. Supplementary Table 3.docx Associations between different lifestyles and all-cause mortality of different age groups after excluding participants who died within the first two years of follow-up. Supplementary Table 4.docx Associations between five healthy lifestyle factors and all-cause mortality of different age groups after excluding participants who died within the first two years of follow-up. Supplementary Figure 1.png The proportion of missing data in the variable. Supplementary Figure 2.png Flowchart for disease trajectory network analyses. Supplementary Figure 3.png Prevalence of 22 chronic medical conditions in 2018. Supplementary Figure 4.png Kaplan–Meier survival curves stratified by lifestyle. Supplementary Figure 5.png Sensitivity analysis of multimorbidity patterns stratified by sex. Supplementary Figure 6.png Sensitivity analysis of multimorbidity networks stratified by sex and lifestyle groups [file 12889_2026_26294_MOESM1_ESM.docx]

**Supplementary Material**

**Healthy Lifestyle, Multimorbidity network, and All-Cause Mortality among Older Chinese: A Longitudinal Analysis in Chinese Longitudinal Healthy Longevity Survey**

Yilin Chen;Huan Zhou;Siqing Wang; Lingqiu Dong;Yi Tang; Jiaxing Tan;Wei Qin

**Supplementary Methods**

**Disease trajectory analyses**

**Step 1**: The first step is to pre-select disease 1 (D1) and disease 2 (D2) pairs with considerable comorbidity strength. First, all possible disease pairs were constructed using the selected 19 chronic diseases using “visNetwork” and “igraph” in R. Then, If the co-occurrence of two chronic diseases in the sub-cohort is at least 1%, relative risk (RR) and Φ-correlation were calculated using the following formulas[1]:

$${RR}_{ij}=\frac{C_{ij}N_{ij}}{C_{i}C_{j}}$$

$$\Phi_{ij}=\frac{C_{ij}N_{ij}-C_{i}C_{j}}{\sqrt{C_{i}C_{j}(N_{ij}-C_{i})(N_{ij}-C_{j})}}$$

Where $C_{ij}$ is the number of individuals affected by both chronic diseases and $N_{ij}$ is the number of individuals in the sub-cohort, while $C_{i}$ and $C_{j}$ are the number of individuals affected by chronic diseases 1 and 2 respectively. For both RR and Φ-correlation measures, the significance of RR=0 and Φ=0 can be both determined using z-test (given large sample size in our study). The corresponding z-score for RR and Φ-correlation were calculated using the following formulas [1, 2]:

$$z_{ij}^{\mathrm{RR}}=\frac{ln({RR}_{ij})}{\sqrt{\frac{1}{C_{ij}}-\frac{1}{N_{ij}}+\frac{1}{C_{i}C_{j}/N_{ij}}-\frac{1}{N_{ij}}}}$$

$$z_{ij}^{\Phi}=\frac{\Phi_{ij}\sqrt{max(C_{i},C_{j})-2}}{\sqrt{1-{\Phi_{ij}}^{2}}}$$

P-values were then calculated using the z-score and adjusted for the issue of multiple testing. Disease pairs with considerable comorbidity strength, representing as significant relative risk (RR) >1.0 and Φ-correlation >0 (i.e., q-value <0.05 for both measures), were eligible for the next step.

**Step 2**: Starting from the pre-selected diseases pairs, the second step aims to identify disease pairs with significant temporal orders (D1→D2):Binomial test was conducted for each disease pair, to investigate whether significantly more individuals had D2 diagnosed after D1 (vice versa) among individuals affected by both medical conditions in the corresponding sub-cohort.

**References:**

1. Hidalgo CA, Blumm N, Barabási A-L, Christakis NA. A dynamic network approach for the study of human phenotypes. PLoS Comput Biol. 2009;5:e1000353.

2. Hou C, Zeng Y, Chen W, Han X, Yang H, Ying Z, et al. Medical conditions associated with coffee consumption: Disease-trajectory and comorbidity network analyses of a prospective cohort study in UK Biobank. The American Journal of Clinical Nutrition. 2022;116:730–40.

**Supplementary Table 1**. Global network metrics stratified by lifestyle group

| Metric | Favourable (n=3,046) | Unfavourable (n=1,878) |
| --- | --- | --- |
| Number of nodes | 14 | 9 |
| Number of edges | 36 | 7 |
| Network density | 0.198 | 0.097 |
| Mean degree | 5.14 | 1.56 |
| Mean out-degree | 2.57 | 0.78 |
| Sum of edge weights | 92.27 | 20.27 |
| Weighted density | 0.507 | 0.281 |

**Supplementary Table 2**. Node-level centrality measures for key diseases in favourable and unfavourable lifestyle groups

| Node | Indegree | Outdegree | Total degree | Weighted indegree | Weighted outdegree |
| --- | --- | --- | --- | --- | --- |
| Favourable group |  |  |  |  |  |
| Hypertension | 1 | 10 | 11 | 1.61 | 24.36 |
| Arthritis | 0 | 10 | 10 | 0 | 18.52 |
| Heart disease | 1 | 6 | 7 | 3.58 | 17.11 |
| Prostate tumor | 2 | 3 | 5 | 3.38 | 12.99 |
| Cataract | 3 | 2 | 5 | 6.72 | 6.32 |
| Diabetes | 2 | 1 | 3 | 4.85 | 3.94 |
| Respiratory disease | 3 | 2 | 5 | 5.2 | 3.65 |
| CVD | 5 | 1 | 6 | 12.43 | 3.43 |
| Ulcer | 1 | 1 | 2 | 2.95 | 1.95 |
| Blood disease | 5 | 0 | 5 | 15.6 | 0 |
| Dementia | 4 | 0 | 4 | 8.94 | 0 |
| Hepatitis | 4 | 0 | 4 | 17.12 | 0 |
| Biliary tract disease | 4 | 0 | 4 | 8.28 | 0 |
| Glaucoma | 1 | 0 | 1 | 1.6 | 0 |
| Unfavourable group |  |  |  |  |  |
| Hypertension | 0 | 4 | 4 | 0 | 13.33 |
| Arthritis | 0 | 2 | 2 | 0 | 4.12 |
| CVD | 1 | 1 | 2 | 3.77 | 2.82 |
| Heart disease | 1 | 0 | 1 | 2.87 | 0 |
| Prostate tumor | 1 | 0 | 1 | 1.54 | 0 |
| Cataract | 1 | 0 | 1 | 2.4 | 0 |
| Blood disease | 1 | 0 | 1 | 5.16 | 0 |
| Respiratory disease | 1 | 0 | 1 | 1.72 | 0 |
| Dementia | 1 | 0 | 1 | 2.82 | 0 |

**Supplementary Table 3.** Associations between different lifestyles and all-cause mortality of different age groups after excluding participants who died within the first two years of follow-up

| **Cox proportional-hazards model^a^** | | | |
| --- | --- | --- | --- |
|  | Exposure | HR (95%CI) | P value |
| **All ages** |  |  |  |
| All-cause mortality | Favourable | 1.0 |  |
|  | Average | 1.26(1.17-1.37) | **<0.001** |
|  | Unfavourable | 1.45(1.31-1.61) | **<0.001** |

^a^ Model was adjusted for age,sex,residence,education,marital status,income, disability at baseline.

**Supplementary Table 4.** Associations between five healthy lifestyle factors and all-cause mortality of different age groups after excluding participants who died within the first two years of follow-up

|  | Exposure | HR (95%CI) |
| --- | --- | --- |
| **All ages** |  |  |
| All-cause mortality | Social engagement(Inactive vs Active) | 1.25 (1.17–1.33) |
|  | Exercise(Non-Current exerciser vs Current exerciser) | 1.04 (0.98–1.11) |
|  | Smoking(Current smoker vs Non-Current smoker) | 1.15 (1.07–1.24) |
|  | Drinking(Current drinker vs Non-Current drinker) | 1.05 (0.98–1.13) |
|  | Diet(Unfavourable vs favourable) | 1.09 (1.02–1.16) |

^a^ Model was adjusted for age,sex,residence,education,marital status,income,state of disability and other 4 factors at baseline.

**
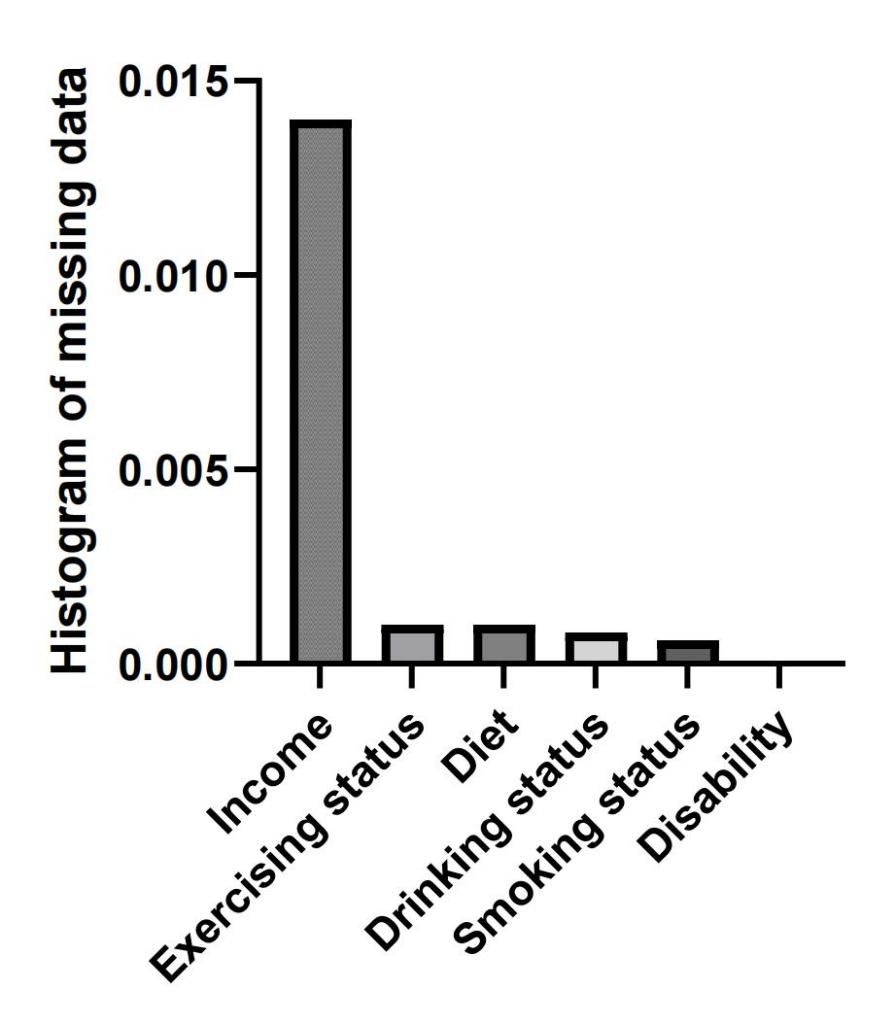
**

**Supplementary Figure 1** The proportion of missing data in the variable

**
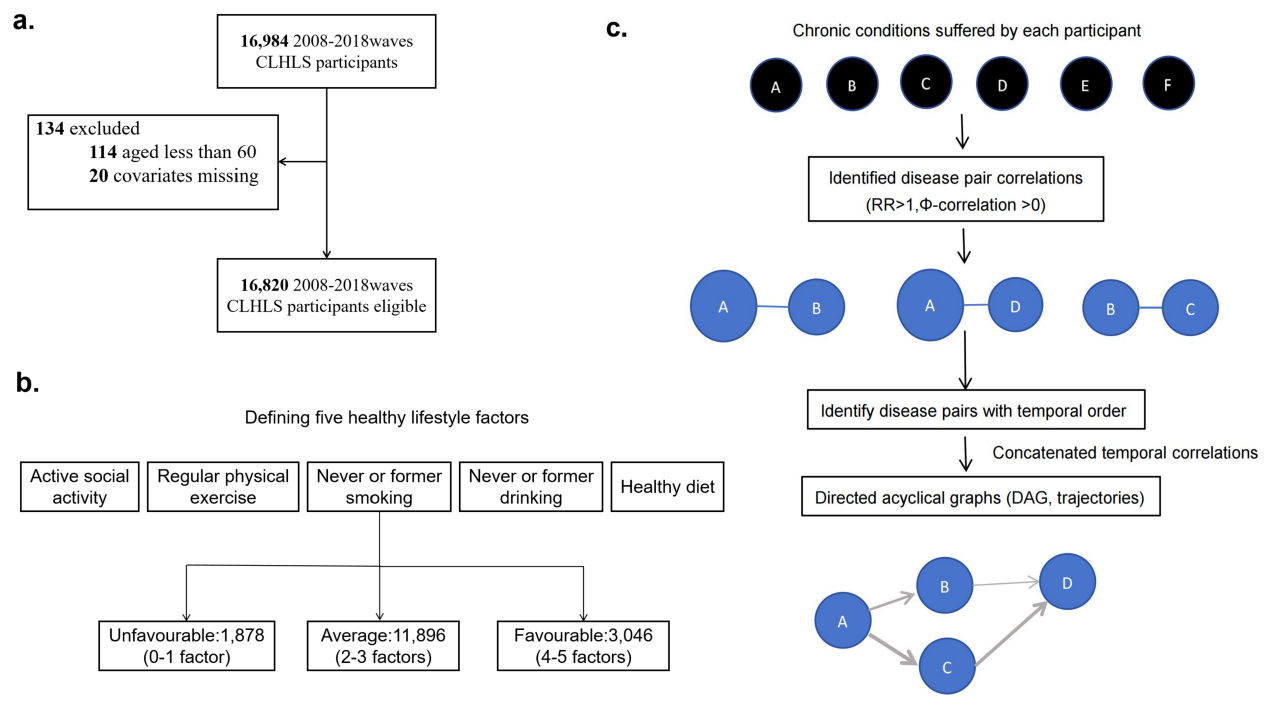
**

**Supplementary Figure 2** Flowchart for disease trajectory network analyses


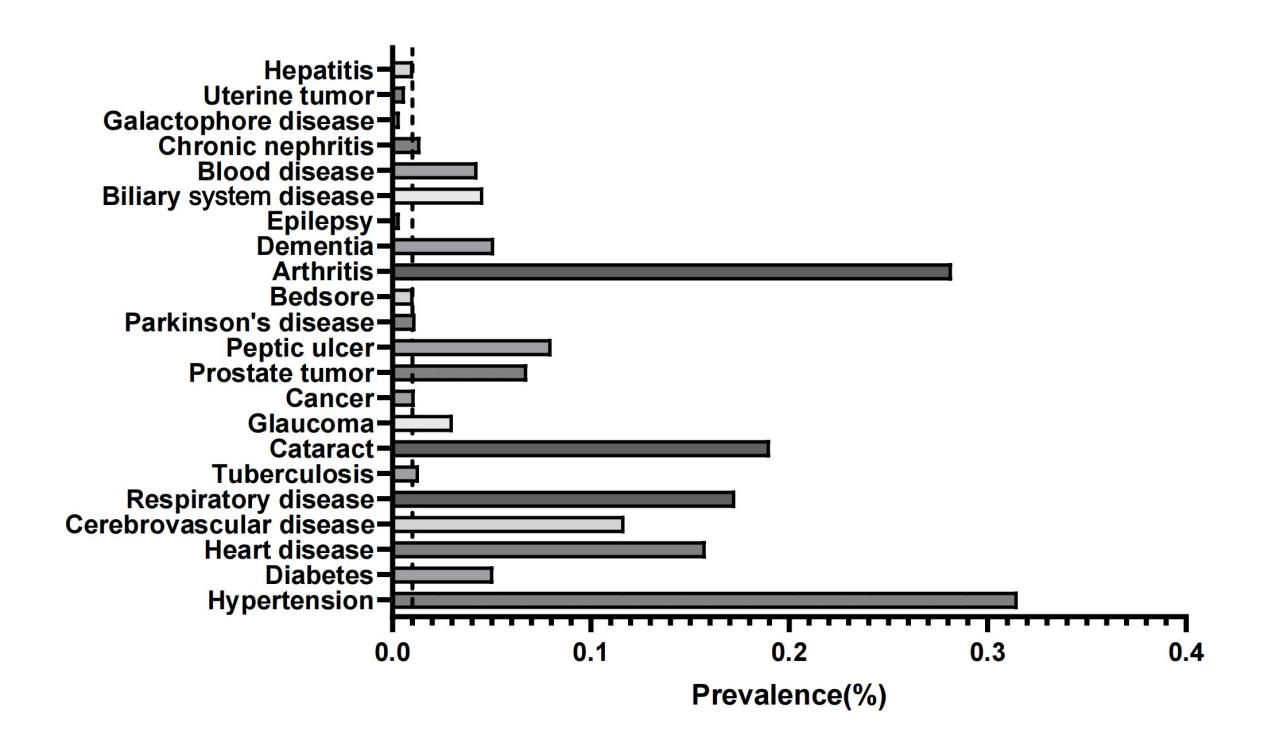


**Supplementary Figure 3** Prevalence of 22 chronic medical conditions in 2018


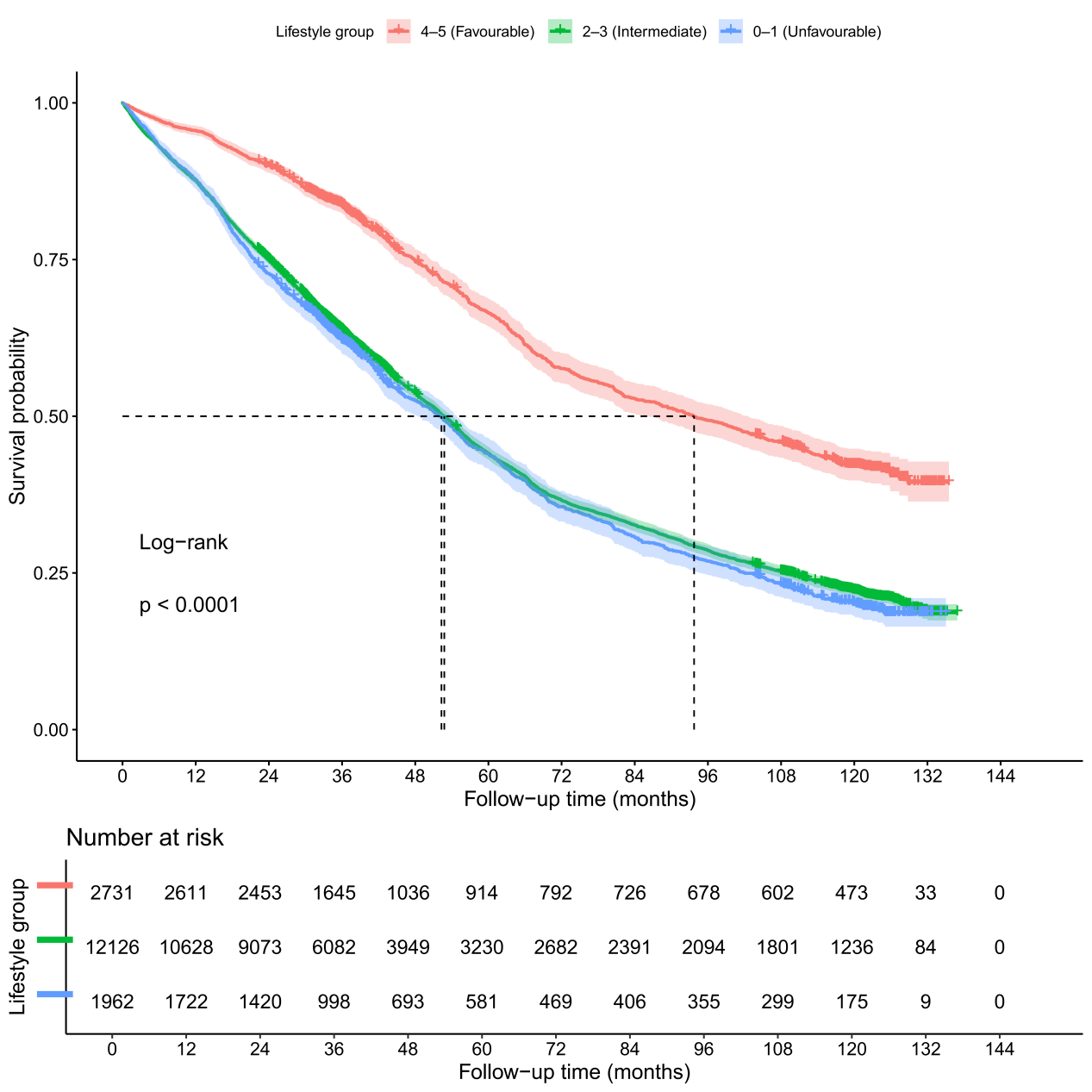


**Supplementary Figure 4** Kaplan–Meier survival curves stratified by lifestyle


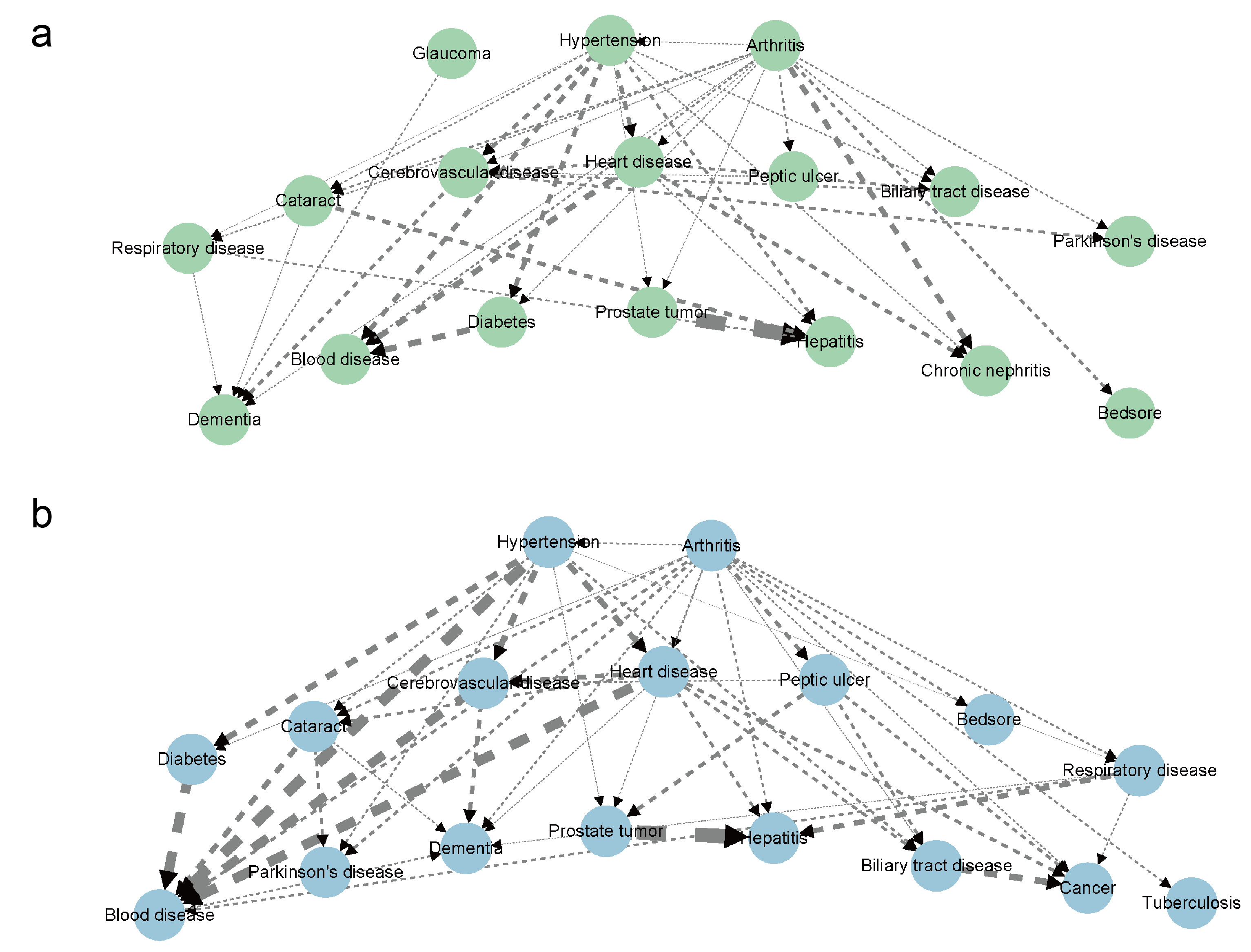


**Supplementary Figure 5** Sensitivity analysis of multimorbidity patterns stratified by sex. (a) Directed disease trajectory network among male participants. (b) Directed disease trajectory network among female participants. Nodes represent diseases, with size proportional to prevalence in each group. Arrows indicate the predominant temporal order of disease occurrence between disease pairs, and edge thickness corresponds to odds ratios obtained from logistic regression models.


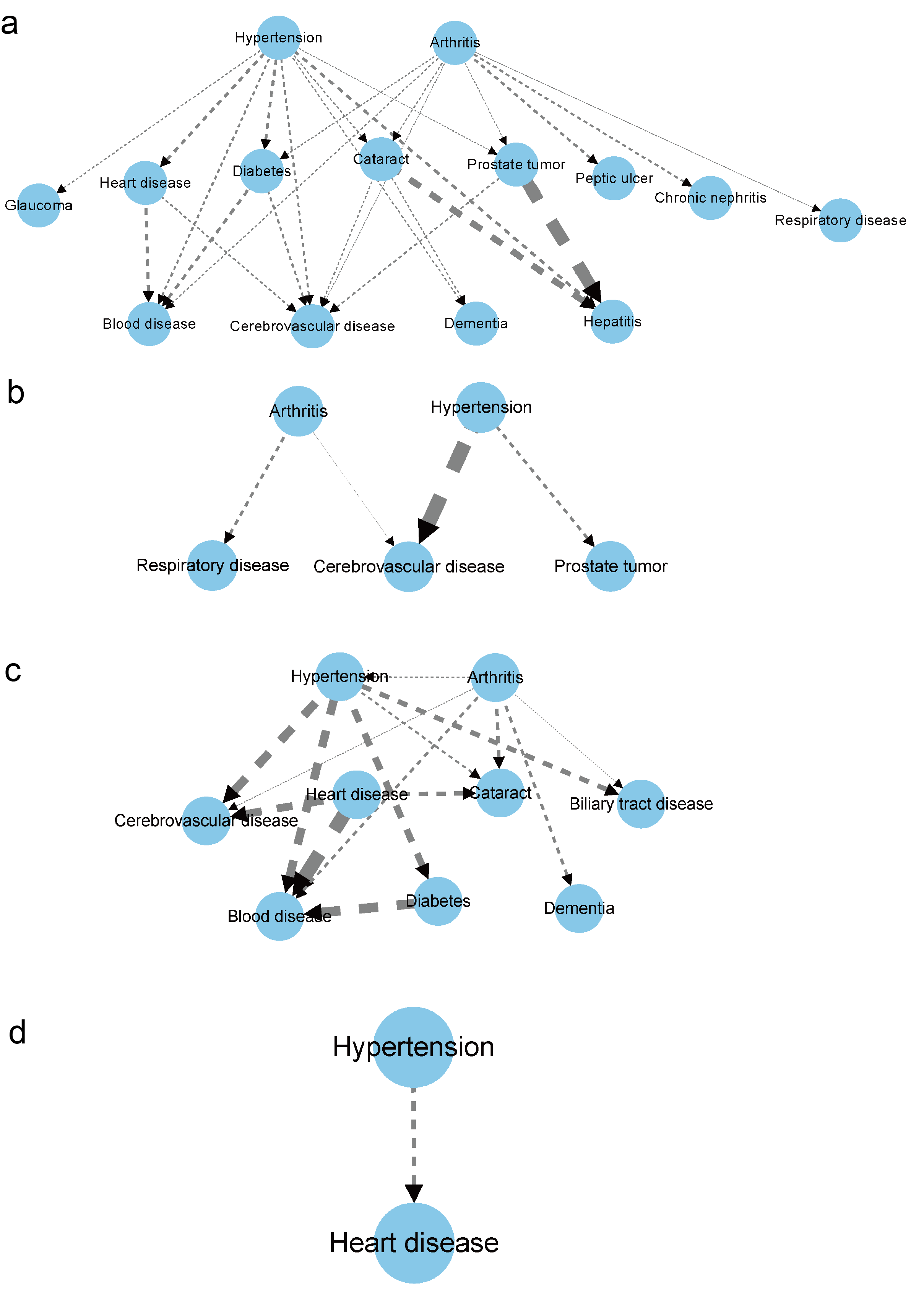


**Supplementary Figure 6** Sensitivity analysis of multimorbidity networks stratified by sex and lifestyle groups. (a) Male participants in the favourable lifestyle group. (b) Male participants in the unfavourable lifestyle group. (c) Female participants in the favourable lifestyle group. (d) Female participants in the unfavourable lifestyle group. The nodes represent chronic diseases, with the size of the nodes proportional to their prevalence in each group. Arrows indicate the predominant temporal order of disease occurrence between disease pairs, and edge thickness reflects odds ratios (ORs) from logistic regression models. The dashed lines represent the lower ORs, while thicker solid lines correspond to higher ORs.
